# Enhancing Antiretroviral Therapy Initiation for Hospitalized and Recently Discharged People Living With HIV in Johannesburg, South Africa

**DOI:** 10.9745/GHSP-D-24-00017

**Published:** 2025-08-14

**Authors:** Natasha Davies, Melanie Bisnauth, Kate Rees

**Affiliations:** a Anova Health Institute, Johannesburg, South Africa.; b Department of Community Health, School of Public Health, University of the Witwatersrand, Johannesburg, South Africa.

## Abstract

**Background:**

Despite increased antiretroviral therapy (ART) access in South Africa, HIV testing and ART initiation are suboptimal in hospital settings. Key gaps include in-hospital case finding, ART initiation support, and primary health care (PHC) facility linkage after discharge.

**Intervention Development and Description:**

We identified weaknesses in hospital processes by comparing them with PHC HIV services and developed a quality improvement model for implementation in 5 Johannesburg hospitals. We introduced dedicated teams of HIV testing counselors for structured case finding and ART-trained nurses and linkage officers to provide in-hospital or post-discharge ART initiation and support to strengthen PHC facility linkage. Monitoring data (May 2020–March 2021) was used to measure initiation rates.

**Lessons Learned:**

Over 11 months, despite COVID-19 pandemic-related disruptions, our model achieved 74% (5,201/7,025) ART linkage within 28 days post-discharge and 87% (6,087/7,025) overall, including all initiations (i.e., all newly diagnosed, known not on ART and reinitiating individuals). The 2 highest-performing hospitals achieved 97% (2,096/2,170) linkage overall, demonstrating the potential of implementing this quality improvement model with fidelity. Over half (58%, 4,092/7,025) of patients initiated ART within 7 days, with 39% (2,748) initiating on the same day. Women and men achieved similar initiation rates (3,010/4,015, 75%; 2,186/3,003, 73%, respectively). Combining rapid (<7 days) in-hospital ART initiation with 28-day post-discharge follow-up supported high ART initiation rates. Using the model mitigated initiation gaps for men and older people, engaging stakeholders supported implementation, and using a team-based approach founded on clear roles and responsibilities improved service delivery.

**Conclusion:**

This model achieved above-average ART linkage rates in a large hospitalized population. We recommend considering introducing this model or adaptations of it to hospitals across South Africa and similar settings where hospital-to-PHC ART service gaps are identified to optimize case finding, ART initiation, and post-discharge linkage support.

## Abstract (precis)

A dedicated HIV-services team can support effective antiretroviral therapy (ART) initiations for hospitalized people with HIV through structured case finding, ART initiation, and post-discharge linkage support. Replicating this model across South Africa and the region could improve individual outcomes.

## Teaser

**Key Findings:**

- Using a quality improvement approach, we implemented and refined a structured intervention that mirrored primary health care (PHC) staff roles to improve HIV case finding, ART initiation, and post-discharge support for hospitalized patients with HIV.
- Over 11 months, we achieved an ART initiation rate of 74% (in-hospital and within 28 days of discharge), well above the global average of 42%. The model effectively closed the gender gap, with males and females achieving comparable initiation rates.
- Regular review meetings ensured teams received implementation support and enabled critical analysis of monitoring data and performance.

**Key Implications:**

- Hospitals are a key entry point for individuals requiring ART, with many individuals with advanced HIV newly diagnosed or re-engaging in care during hospital admission. Policymakers and managers should review hospital-based HIV service structures and protocols, ensuring clearly defined roles and responsibilities, strengthening communication pathways, and developing mechanisms to safeguard linkage between hospital and PHC facilities after discharge.
- Policymakers, managers, and national stakeholders should prioritize addressing acknowledged weaknesses in hospital-based HIV services and consider implementing a quality improvement model to ensure effective HIV services are established in high-burden countries.

## BACKGROUND

South Africa has a high HIV prevalence of 13.3% and provides the largest ART program globally.^1^^,^^2^ In 2004, when South Africa’s national ART program was launched, ART provision was limited to hospital settings, hindering access.^2^^,^^3^ Consequently, in 2010, South Africa decentralized HIV services, using task-shifting to expand ART provision to primary health care (PHC) facilities through nurse-driven ART initiation and management (NIMART). The NIMART nurse cadre was limited to PHC settings.^4^^,^^5^ This created different service delivery approaches between PHC facilities and hospitals. At PHC facilities, clear roles and responsibilities emerged with lay counselors providing HIV testing and counseling services (HTS), NIMART nurses providing ART initiation and management services, and lay counselors, sometimes known as linkage officers, supporting patient adherence, treatment literacy, and retention in care.^2^ Although there is a dearth of published literature specifically addressing the design of hospital HIV services, based on the authors’ extensive programmatic experience, HIV services in South African hospitals continue to be doctor driven, guided by hospital-specific policies and protocols that preclude nurses from providing HIV and ART services. Many hospitals have poorly developed HIV-related policies and ill-defined doctor roles and responsibilities.^6^ In South Africa, hospital HIV services are typically provided through 1 of 2 models: (1) nonspecialist doctors provide services according to their knowledge of and confidence in applying existing ART guidelines, or (2) all clients are referred to a specialist infectious diseases team for ART initiation. These models limit the reach of HIV testing and ART initiation support in hospitals, particularly in nonmedical wards (e.g., surgical and gynecology wards), leaving considerable coverage gaps.

HIV services in South African hospitals continue to be doctor driven, guided by hospital-specific policies and protocols that preclude nurses from providing HIV/ART services.

South Africa implemented universal test and treat and same-day initiation policies in 2016 and 2017, respectively.^7^^–^^9^ These policies were implemented across the PHC program through wider availability of counselor-driven rapid HIV testing services, provider-initiated counseling and testing, and NIMART nurses. These policies and other interventions have expanded ART coverage to more than 5 million South Africans^1^ and have driven declining HIV-related deaths over the past decade.^10^^–^^14^ Contrastingly, within hospitals, testing continued to rely heavily on lab-based diagnostic testing with slow turnaround times, making same-day initiation difficult. Additionally, advanced HIV disease (CD4 count <200 cells/m^3^) remains prevalent in South Africa, affecting 10%–15% of newly diagnosed people living with HIV (PLHIV), many of whom are newly identified during hospital admission.^11^^,^^12^^,^^14^ Up to 40% of hospitalized patients are ineligible for same-day ART initiation because of complex comorbidities and lack of psychological readiness.^14^^–^^16^ Poorly established referral pathways between hospitals and PHC facilities compound loss to follow-up, morbidity, and mortality by hindering post-discharge ART continuation or initiation in deferred patients.^10^^,^^14^^,^^15^^,^^17^^,^^18^ By 2018, with increasingly progressive policies, ART linkage at the PHC level in Johannesburg improved to 88%, but hospitals lagged behind, averaging only 43% ART linkage (unpublished data, DHIS 2018).

Inconsistent use of standardized clinical stationery, registers, and electronic health information systems that did not interface with the established national HIV database (TIER.Net) further exacerbated hospital service deficiencies. Patients who did engage with HIV care during hospital admission often remained “invisible” within the national database, with no means of tracking whether they remained in care at the PHC level post-discharge. Inadequate data systems also resulted in ineffective performance monitoring for hospital HIV services.

A recent systematic review highlighted a 26% mortality rate among hospitalized individuals within 6 months post-discharge, emphasizing the need for interventions to strengthen early post-discharge follow-up to reduce readmissions, morbidity, and mortality.^14^^,^^19^ Such poor outcomes are particularly important for men who continue to be disproportionately affected by late HIV diagnosis, advanced HIV disease, and lower ART initiation rates.^20^^,^^21^ Because of these gender-based discrepancies in the HIV care continuum, men—both newly diagnosed and disengaged from care—have been identified as an important target group for HIV testing, ART linkage, and support in hospital settings.^22^^–^^24^

To address the disparities between PHC facility-based and hospital-based HIV services, we designed a quality improvement intervention to strengthen in-hospital case finding, ART initiation, and post-discharge linkage support.

## INTERVENTION TO IMPROVE THE QUALITY OF HIV SERVICES IN SOUTH AFRICAN HOSPITALS

### Setting

We selected the 5 largest hospitals (2 tertiary, 1 regional, and 2 district) in the Johannesburg Health District to conduct our quality improvement intervention. According to program data, at the time of initial implementation in mid-2019, the 5 hospitals had a combined 4,846 in-patient beds, with an average bed utilization rate of 83% and an average admission length of 6 days. In 2020, Johannesburg Health District had a general population HIV prevalence of 13%, with 28% in females aged 25–49 years and 15% among those aged older than 50 years. Johannesburg Health District had reportedly achieved an overall 86%, 78%, and 84% for the Joint United Nations Programme for HIV/AIDS 95/95/95 HIV care continuum targets.^25^

### Intervention Development

#### Step 1: Compare Primary Health Care Facility and Hospital-Based HIV Services

We explored the underlying reasons for discrepancies in performance between PHC facility and hospital-based HIV services by comparing these services to identify key gaps and differences. First, we undertook a desk-based review of hospital and PHC facility policies and program documents. Second, we observed HIV-related client flow and staff roles at several PHC facilities in Johannesburg and the 5 chosen hospitals. We sought to understand how HIV services were structured, which staff cadres were responsible for each component of the service, and how the services were being monitored (Table 1).

**TABLE 1.** Comparison of Standard Practice for PHC-Based HIV Services, Pre-Intervention, and Post-Intervention Hospital HIV Services, Johannesburg, South Africa

	**PHC Facilities**	**Before Intervention: Hospitals**	**After Intervention: Hospitals**
HTS	Lay counselors provide HTS Nurse clinicians offer PICT to all HIV negative/unknown status PHC clients Point-of-care rapid antibody tests with immediate results communicated to client HIV tests and results recorded in national HIV testing register	Doctor-driven PICT inconsistent, particularly in nonmedical wards, may order lab-based ELISA Lay counselors conduct rapid HIV testing only on doctor referral Results often unavailable before discharge ELISA results not captured in register	Lay counselor-driven Daily counselor ward rounds for new admissions Bedside rapid point-of-care antibody HIV tests Immediate communication of results to patient and NIMART nurse HIV test and result recorded in national HIV testing register
ART initiation	Lay counselors refer all clients to facility NIMART for same day initiation if eligible Standard clinical stationery used and captured daily onto database (i.e., TIER.Net)	ART initiation dependent on available ELISA result pre-discharge & sufficiently trained/confident doctor No NIMART nurses in hospitals Often deferred for initiation at PHC facility Initiation data often not captured on TIER.Net	Provincial permission for NIMART nurses’ appointment Counselors refer clients for bedside same day ART assessment/ initiation NIMART nurses work with ward doctors Standard clinical stationery used and initiations captured weekly on TIER.Net
Linkage to ART continuation or deferred ART initiation	TIER identifies missed appointment or loss to follow up after initiation Lay counselors provide adherence/treatment literacy sessions Monitoring: follow-up visits on standardized clinical stationery, captured on TIER.Net, enables tracing/recall of missed appointments NAG standardized approach	Inconsistent use of stationery Limited mechanisms to confirm linkage ART continuation post-discharge Poor hospital-PHC facility referral pathways Inconsistent NAG implementation with limited adherence/treatment literacy counseling	New clients referred to linkage officer for NAG -based adherence/treatment literacy counseling Standardized transfer letter from NIMART nurse accompanies discharge summary Linkage officer discusses discharge plan and provides 28-day telephonic follow-up Linkage officer completes 28-day register Data capturer captures transfers on TIER.Net

Abbreviations: ART, antiretroviral therapy; ELISA, enzyme-linked immunosorbent assay; HTS, HIV testing and counseling services; NAG, National Adherence Guidelines, NIMART, nurse initiated and managed ART; PHC, primary health care; PICT, provider-initiated counseling and testing; PLHIV, people living with HIV.

We explored the underlying reasons for discrepancies in performance between PHC facility and hospital-based HIV services.

#### Step 2: Design Improved Hospital HIV Services

Using the service comparison findings, the research team, in collaboration with clinicians and technical advisors at Anova Health Institute and the Department of Health familiar with hospital and PHC services, designed a quality improvement intervention, encompassing components that addressed in-hospital case finding and ART initiation, as well as post-discharge linkage. The research team presented the proposed model during numerous stakeholder engagements and discussions with district and hospital management staff. The Gauteng Provincial Department of Health was also approached to approve NIMART nurse initiation and prescription of ART in hospitals within the province, including the 5 selected hospitals. Approval was secured in February 2019.

### Intervention Description

**FIGURE 1:**
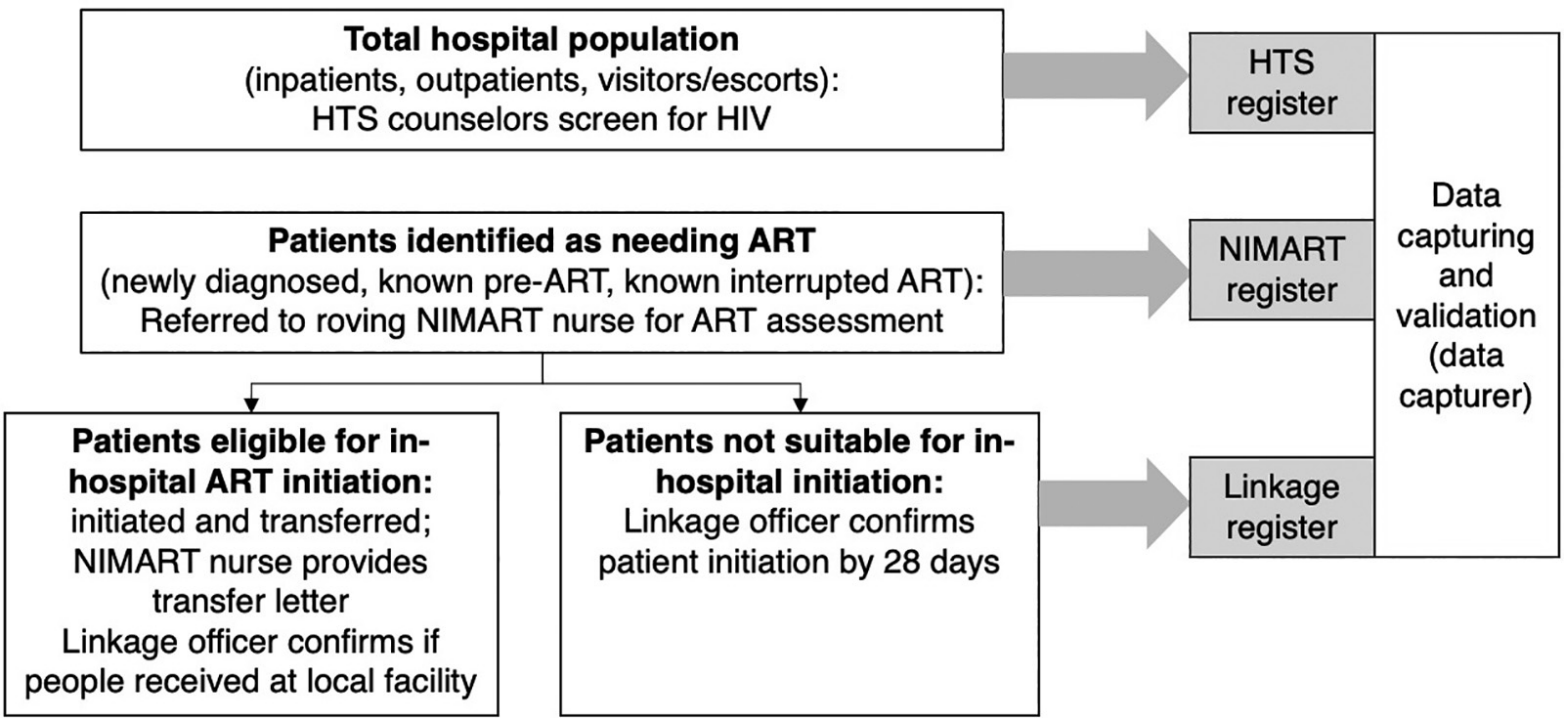
In-hospital Case Finding and Antiretroviral Therapy Initiation, Johannesburg, South Africa

We developed a dedicated HIV-services team comprising HTS counselors, NIMART-trained nurses, and linkage officers (lay counselors) and clearly defined team member roles and responsibilities adapted from those observed within PHC facilities (Figure 1). The HTS counselors were responsible for case finding and conducted daily reviews of all new admissions across all medical and nonmedical wards to identify patients with unknown HIV status for testing, as well as known people with HIV but not yet or previously on ART for (re)initiation. Visitors and escorts were also screened where possible; however, due to time constraints, coverage of these groups was low.

The NIMART nurses facilitated in-hospital ART initiation. All ART-requiring patients were immediately referred to the NIMART nurses for bedside clinical and psychological readiness assessment and same-day ART initiation or deferral (Figure 1). For the first time, NIMART nurses were introduced to the 5 hospitals. To enhance collaboration and facilitate comprehensive patient care, these newly appointed NIMART nurses worked alongside existing ward doctors, and efforts were made to establish positive relationships between the HIV team and ward-based clinical teams. When ART initiation involved complexities (e.g., renal dysfunction), doctors provided clinical guidance and supported decision-making.

**FIGURE 2:**
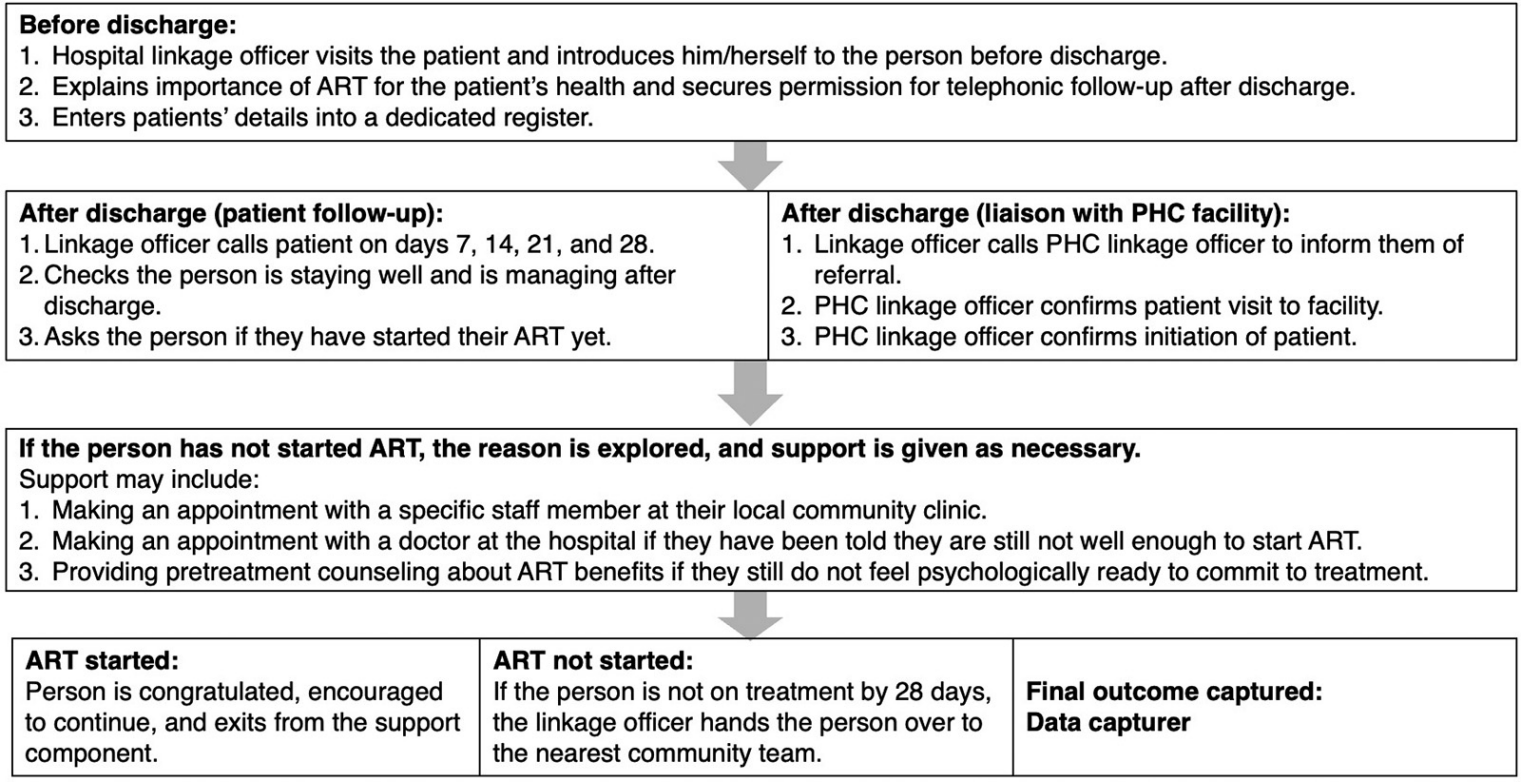
Post-Discharge 28-Day Follow-Up Component, Johannesburg, South Africa

The linkage officers provided in-hospital treatment literacy counseling and ensured post-discharge follow-up and linkage to PHC clinics. Simultaneous liaison with linkage officers ensured ART-initiated patients experienced smooth transfer to their chosen PHC facility for continued management of ART and comorbidities post-discharge (Figure 2).

For patients who had been initiated on ART, the communication pathway and referral mechanisms between hospital and PHC facilities were strengthened in numerous ways. Upon discharge from the hospital, patients who had been initiated on ART received a standardized Department of Health transfer letter from the NIMART nurse and a treatment literacy counseling session from the hospital linkage officer to encourage ART continuation at their chosen facility. Then, to facilitate a smooth transition from hospital to PHC facility, the hospital linkage officer made a single call to the receiving PHC linkage officer to alert them to expect the new patient and ensure patients were successfully linked to their designated local facility after discharge. Once the PHC linkage officer confirmed that the patient had been received at the PHC clinic, ongoing support was handed over to the PHC facility team, and the patient exited the intervention. Linkage officers liaised with on-site data capturers to ensure all clients were captured in the national ART database, TIER.Net, to enable tracking after discharge.

For those clients who were ineligible for in-hospital ART initiation, the 28-day post-discharge support component was activated (Figure 2), assisting referral to local PHC facilities for timely ART initiation. The linkage officer verified every post-discharge ART initiation using the following procedure. First, the hospital linkage officer called the patient every 7 days for 28 days after discharge to confirm if they had initiated ART and encouraged and assisted them to initiate ART if they had not yet done so. When the patient self-reported ART initiation, the hospital linkage officer called the PHC facility linkage officer to confirm initiation captured on TIER.Net. If the patient was not found on TIER.Net, the hospital linkage officer called the patient to investigate and encourage ART initiation. Patients not initiated by 28 days were actively handed over to PHC facility teams for ongoing support and follow-up before being removed from the hospital linkage officers’ deferred-ART patient list.

### Implementation

In mid-2019, we launched implementation in the 5 hospitals. In the first 12 months, hospital-based HIV teams were appointed, and the new teams established positive relationships with existing hospital staff. Toward the end of the first year of implementation, the COVID-19 pandemic hit South Africa, and the first national lockdown occurred in March 2020. The teams remained in place, continuing model implementation within a rapidly shifting hospital environment.

For the first year of implementation, hospital teams conducted monthly review meetings. Challenges with the model, areas for improvement, and best practices were discussed to ensure model adjustments occurred across all sites. A crucial component of this monthly review process was refining reporting tools so that teams could focus on recording data that was useful clinically and for the intervention, minimizing their administrative burden and optimizing efficiency. The PDSA (plan, do, study, act) quality improvement approach was used to gradually refine, adjust, and adapt the model based on critical data analysis and feedback from team members.

Each step was essential to the model’s integrity and triggered the initiation of subsequent steps. Daily communication among team members ensured updates on client progress, identifying which steps were outstanding. Weekly, the 3 registers (HIV testing, NIMART, and linkage) were compared to track individuals’ movement through the system, with confirmation of ART linkage and accurate data capture in TIER.Net. Inconsistent implementation of steps, especially because of gaps in hospital management support or staffing, particularly NIMART nurses, negatively impacted initiation rates, as seen in the lowest-performing hospitals.

## INTERVENTION MONITORING

We analyzed data for the period after the model had been refined—during the peak of the COVID-19 pandemic-—between May 2020 and March 2021. Strengthening routine data was a key component of the quality improvement model. Dedicated, on-site data capturers liaised weekly with clinical teams to collect and validate monitoring data from HTS counseling registers, NIMART nurse assessment and initiation registers, and linkage officer post-discharge tracking registers. Registers collected gender, date of birth, HIV test date and result, and ART initiation date or alternative 28-day outcome (lost to follow-up, died, or transferred). Transferred was defined as the patient requesting relocation to another facility with handover of care before ART initiation. Lost to follow-up was defined as unable to make telephonic contact with the patient after 3 attempts (wrong number, no answer, or voicemail). Data capturers entered the data weekly into an electronic database from which monthly reports were compiled. Monitoring and evaluation supervisors oversaw data quality and conducted validation activities. Implementation teams held monthly meetings during which monthly reports were reviewed, any data queries addressed, and final numbers validated to confirm overall ART initiation rates.

For this analysis, we used 11 months of routine monitoring data from May 2020 to March 2021 once the initial design, planning, and modification phases of implementation had been completed and the model had been optimized. During these 11 months, individual-level routine data were collected on standardized reporting tools. Our primary indicator was ART initiation rate (total ART initiated by 28 days post-discharge/total number identified needing ART). Our secondary indicator was time to initiation based on the duration between the date identified as requiring ART (from HTS register and NIMART nurse assessment register) and the ART (re)initiation date (from the NIMART nurse register or linkage officer register after TIER.Net verification). We disaggregated these indicators by age and gender. Data analysis was undertaken using Excel.

Additionally, for 4 of the 5 hospitals, we report data from the DHIS, the national system used to report aggregated indicators. The fifth hospital uses a different information system from which we could not access data. The DHIS indicators differ from our intervention indicators in that only newly diagnosed, first-time initiations were included, with no capturing of pre-ART or reinitiating clients. There were also data quality concerns with these indicators for hospitals because of challenges associated with monitoring, evaluation, and reporting processes within hospital services. We analyzed linkage rates (number of new ART initiations/number of positive HIV tests) in 3 periods: 6 months pre-intervention (October 2018–March 2019), 12 months of early implementation (before the COVID-19 pandemic, April 2019–March 2020), and 12 months of full implementation (during the COVID-19 pandemic April 2020–March 2021). The 11 months of monitoring data fall during the full implementation period. The DHIS data are captured as aggregated data elements and exclude post-discharge ART initiations, leading to an expected discrepancy with our data. We believe our data offer a more complete picture as it tracks individuals over time, including post-discharge. However, because data of this type were unavailable before the intervention began, we used DHIS data to demonstrate changes before and after intervention implementation.

### Ethics Approval

Site-level monitoring data was de-identified before analysis. Ethics approval was granted by the Human Sciences Research Council (HSRC), approval number REC 3/22/08/18.

## LESSONS LEARNED

### Combining In-Hospital Antiretroviral Initiation With Post-Discharge Follow-Up Supported High Antiretroviral Initiation Rates

**FIGURE 3:**
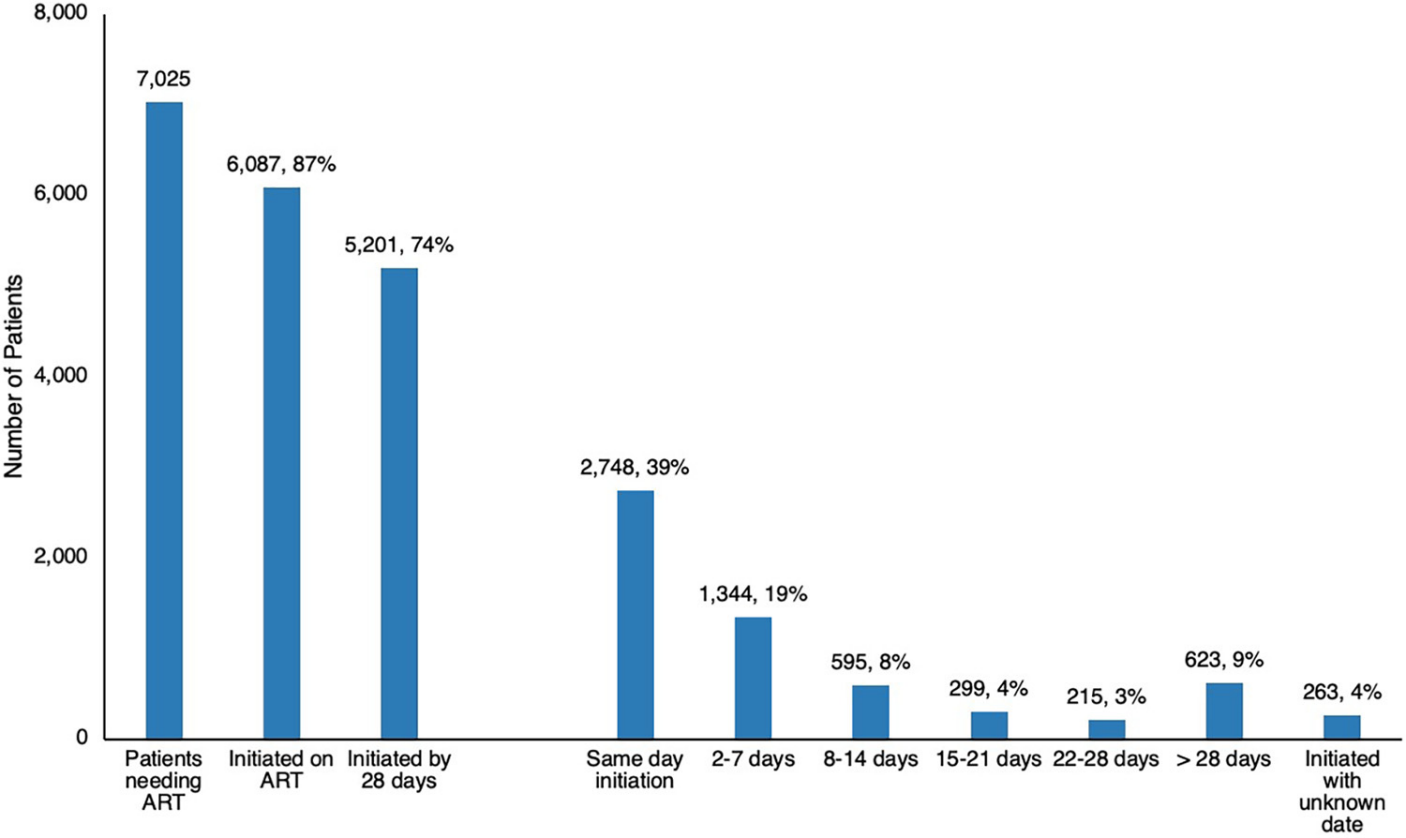
Time to ART Initiation in Implementing Hospitals, Johannesburg, South Africa, May 2020 to March 2021 Data source: Intervention data.

Between May 2020 and March 2021, the teams identified 7,025 patients requiring ART (re)initiation across the 5 hospitals. Of these, 58% (4,092) initiated within 7 days, including 39% (2,748) with same-day initiation, 74% (5,201) initiated ART by 28 days of follow-up (Figure 3), 9% (623) initiated beyond 28 days, and 4% (263) initiated with an unconfirmed date. Overall, the model achieved 87% (6,087/7,025) linkage to ART with 13% (938) not confirmed initiated on ART.

Of the 7,025 patients identified as requiring ART in the 5 hospitals, 6,087 were initiated on ART.

A total of 198 clients died before ART could be initiated, representing a known mortality rate of 2.8%. These mortality data are based on information gathered by linkage officers post-discharge, so the data are likely an underrepresentation only describing known mortality within the 28-day follow-up period. Other reasons for not initiating ART, including transfer out and loss to follow-up, were not well captured.

**FIGURE 4:**
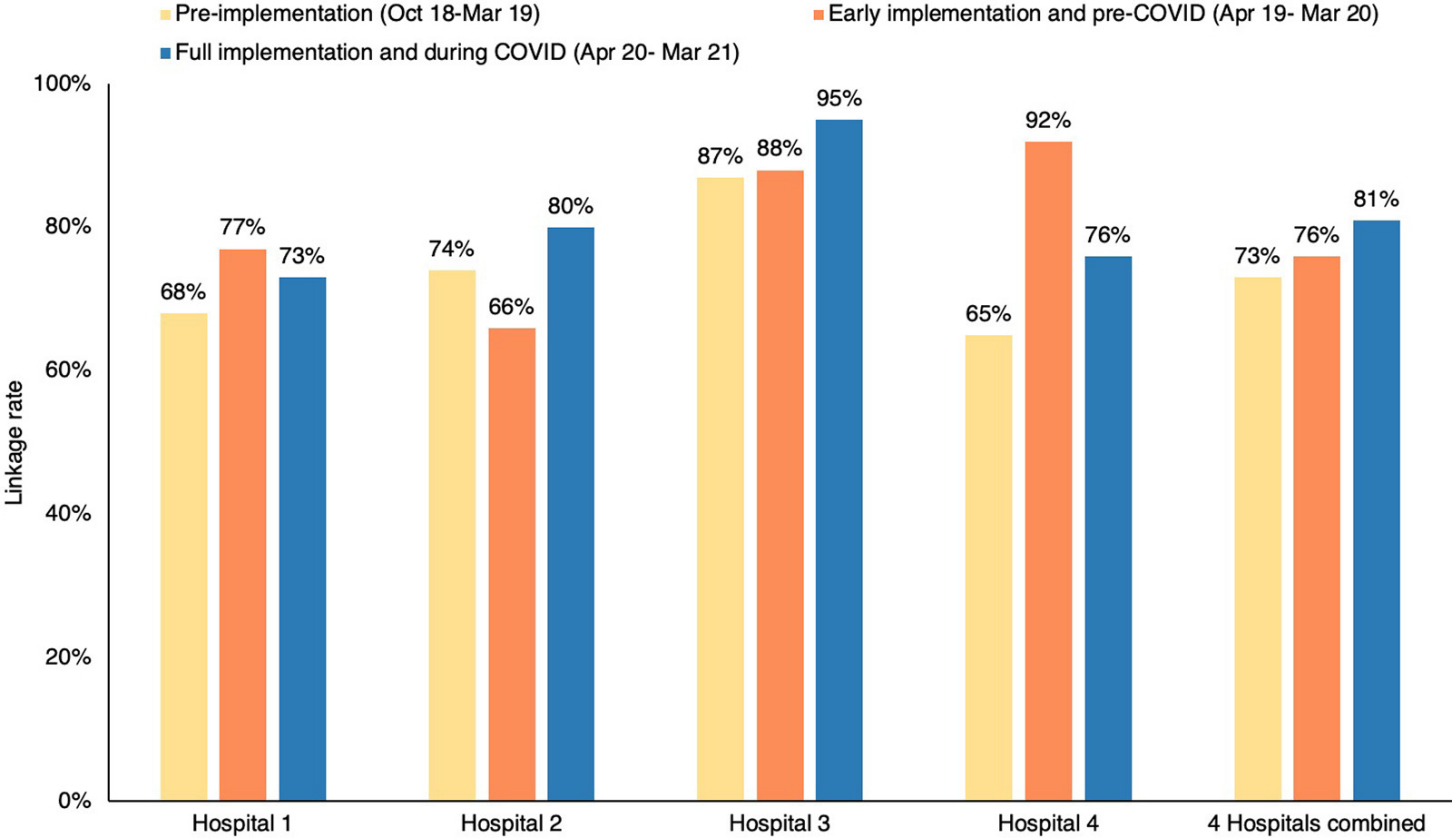
ART Linkage Rate Pre-implementation, Early Implementation, and Full Implementation, Johannesburg, South Africa Data source: DHIS.

With full fidelity of all model components demonstrated by the 2 highest-performing hospitals, an 84% ART initiation rate for 28 days and an overall 97% initiation rate were achieved (intervention data). However, 2 hospitals achieved only a 53% initiation rate because of staff shortages. For prolonged periods during implementation, hospital 5 (not included in Figure 4, DHIS data) lacked NIMART nurses to conduct bedside assessments and initiation for all identified patients. This demonstrated the key role of a full, multidisciplinary team, including the appointment of NIMART nurses within the hospitals.

### The In-Hospital Model Mitigated Typical Initiation Gaps for Men and Older People

Similar proportions of women (87%; 3,507/4,015) and men (86%; 2,575/3,003) initiated ART, including same-day initiation. Of the 6,087 patients who initiated ART overall, 42% (2,575) were men. This is higher than the proportion of positive tests (25%–30%) contributed by male patients accessing HTS in PHC settings (intervention data). This emphasizes that men remain an important target group for HTS and ART linkage and support in hospital settings.^22^^–^^24^

The largest proportion of women (1,339, 33%) and men (1,063, 35%) were aged 35–44 years. However, 34% (2,395) of all patients were aged 45 years or older, highlighting that older age groups should continue to be targeted with services that identify PLHIV not yet engaged in care. This is particularly true for men who often present at older ages and with more advanced HIV.^26^ Forty-one percent (1,231/3,003) of men in this cohort were aged 45 years or older. The HIV program in South Africa, as with many other countries in the region, continues to face gaps in reaching both men and older age groups. By implementing a universal test and treat approach, whereby HTS counselors reviewed every new admission to their hospital, the in-hospital teams were able to identify and link almost equal numbers of men compared to women and a high proportion of individuals aged 45 years or older. These groups may be missed with community and PHC facility-based HIV testing and linkage strategies, emphasizing the importance of strengthening hospital-based HIV services.

By implementing a universal test and treat approach, the in-hospital teams identified and linked almost equal numbers of men compared to women and a high proportion of individuals aged 45 years or older.

### Stakeholder Engagement Crucially Supported Implementation

There were 2 critically important precursors to the successful implementation of this quality improvement model. The first was a careful analysis of why HIV services within the hospitals had not resulted in effective ART linkage compared to PHC services. By conducting a comparison of the hospital and PHC facility-based HIV services, the intervention team identified that a lack of clear roles and responsibilities contributed to inefficiencies and gaps. Second, the analysis findings were shared with key stakeholders during extensive, in-depth discussions with provincial, district, and hospital management. These engagements paved the way for hospital buy-in and smooth implementation processes. It was important to present a persuasive argument for the need for change using the initial analysis and then to work collaboratively using the PHC model to develop a hospital model that managers were satisfied with. High-level stakeholder engagements also facilitated a crucial provincial policy change, permitting the appointment of NIMART nurses who could undertake ART-related clinical activities previously prohibited within hospitals, resulting in successful task-shifting away from doctors.

### Using a Team-Based Approach Founded on Clear Roles and Responsibilities Aided Buy-In

As seen during the PHC program assessment, a valuable component of this intervention was the structured approach facilitated by a dedicated team with clearly defined roles, responsibilities, and clinical support tools. This team fostered connections with other hospital staff, strengthened internal referral systems, and promoted teamwork and awareness of HIV management. Additionally, it alleviated the burden on existing ward staff, who often lacked time, knowledge, and confidence when applying ART guidelines. The constant presence of the HIV teams on the wards and regular clinical discussions with treating clinicians supported increased awareness of the HIV intervention within ward teams.

A valuable component of this intervention was the structured approach facilitated by a dedicated team with clearly defined roles, responsibilities, and clinical support tools.

### Completing Key Steps Maximized Model Benefits

Effective teamwork ensured the completion of all key steps in the linkage support model, each critical for promoting ART initiation and post-discharge continuity of care. Table 2 summarizes the benefits, challenges, and lessons learned from each step.

**TABLE 2.** Benefits, Challenges, and Learnings for Hospital HIV Services Model, Johannesburg, South Africa

Step	Benefits	Challenge/Learning
Department of Health approved transfer letter	Facilitated hospital to PHC transition Communicates medical management to receiving team improving continuity	Few challenges but printed transfer letters must be available Client must be informed that they need to present letter at PHC
Hospital and PHC-based linkage officer liaison	Enabled tracking of mobile individuals after discharge Enabled early identification and intervention for those whose arrival was delayed	Availability of mobile phone data and network High staff turnover at PHCs An electronic referral system could mitigate these
Team interactions with data capturers	Weekly review of 3 registers (HIV testing, NIMART, linkage officer tracking) and use of TIER.Net ensured accurate capturing Accurate data capturing supported quality reporting and evaluation, with rapid identification of gaps	Network connectivity and database downtime leading to back capturing Feedback from PHC linkage officers was often delayed Paper-based and non-integrated systems
Comprehensive 28-day client support	28 days of personalized telephonic follow up by a known linkage officer was the cornerstone of this model Consistent, supportive, individualized follow-up contributed 15% to overall linkage	Incorrect numbers, no answer, network issues often meant individuals could not be reached, even on several attempts In future, with consent, a next of kin or treatment supporter number would be useful AI/chatbot models may be considered for alternative support

### Supportive Supervision, Flexibility, and Dedicated Effort Minimized the Impact of COVID-19 Pandemic Disruptions

Implementation data were collected during the height of the COVID-19 pandemic (May 2020–March 2021). The disruption to health care services, including HIV services, has been extensively documented.^14^^,^^27^ Inevitably, COVID-19 pandemic-related disruptions impacted our teams extensively. Changes to hospital protocols and admission criteria for non-COVID-19-related conditions affected the ability of HTS counselors and NIMART nurses to interact with patients because of restricted movement of staff providing non-COVID-19 services. Hospital areas and wards were divided into different zones based on COVID-19 infection status, including red zones for confirmed COVID-19 cases, orange zones for patients under investigation for COVID-19, and green zones for non-COVID-19 cases. Teams were permitted to continue working in the green zones, which enabled ongoing implementation to a large proportion of hospitalized patients. Some patients in the red and orange zones were managed by COVID-19 ward teams, including HIV testing and initiation, while others were linked to our team once they were moved to green wards. It is not possible to accurately gauge the impact that COVID-related disruptions had on this model; however, considering the extent of the negative impact on health services, including HIV/TB services,^27^^,^^28^ it is striking that this model achieved a 74% initiation rate by 28 days post-discharge, with no decline in ART initiations. Hospital 4 experienced the steepest decline in ART initiations because of strict admission criteria for non-COVID-19 cases, which hindered the team’s ability to link individuals. Additionally, the absence of a NIMART nurse for an extended period further compounded these challenges. In contrast, although hospitals 2 and 3 took longer to implement the full model with fidelity during the pre-COVID-19 pandemic period, they managed to maintain high ART initiation rates despite the numerous challenges posed by the pandemic.

During this time, the monthly team meetings proved invaluable opportunities for debriefing and providing support to the teams. The teams could constantly review COVID-19-related site changes and adapt through supportive guidance from the intervention managers.

Figure 4 presents data from the DHIS on pre-, early, and full implementation linkage rates for 4 hospitals with an overall linkage rate of 81%. The fifth hospital was excluded as it used a nonintegrated reporting system that did not consistently submit to DHIS. Using intervention data, verified monthly, for all 5 hospitals, which included post-discharge linkages, the hospitals achieved an overall linkage rate of 87%.

## SCALABILITY AND RESOURCE IMPLICATIONS

This model could be particularly beneficial in high HIV-burden countries like South Africa by bridging gaps between hospitals and PHC facilities. While it may appear resource-intensive because of the costs, this dedicated team mirrors existing low-cost roles in primary care, such as counselors, linkage officers, and data capturers, resulting in task-shifting at the hospital level. Given the high costs associated with mortality, morbidity, and hospital readmissions because of poor linkage between hospitals and PHC facilities, having a dedicated team, despite limited resources, may be justifiable.

Although developing the model was initially intensive, ongoing input and supervision requirements decreased to standardized monthly reporting, suggesting that with sufficient initial support for hospital management buy-in and relationship building, the model can become sustainable with minimal oversight. An implementation guide was developed to facilitate adoption by other hospitals, bypassing the need for a prolonged quality improvement phase.

However, in many high HIV burden countries facing human resource challenges, implementing a dedicated multidisciplinary team may not be feasible. Alternative adaptations could include training ward-based nurses in NIMART, enhancing collaboration between counselors and doctors for in-hospital ART initiation, assigning a single case manager for post-discharge care, training existing data capturers to engage with national HIV databases, and transitioning to electronic medical records. In the future, components like the 28-day linkage support could be delivered via technology, such as artificial intelligence chatbots, for post-discharge messaging.

Although this model was focused on supporting PLHIV, other chronic conditions, such as diabetes, hypertension, and mental health disorders, are frequently identified during hospital admission and rely on long-term therapy to optimize health outcomes. Expanding the model to support hospitalized individuals with other chronic health conditions could enhance its cost-effectiveness and the integration of chronic disease management. Training the multidisciplinary team to address various chronic conditions may improve outcomes in resource-constrained settings. Future research should assess the cost-effectiveness and broader applicability of this model across different health conditions and resource levels.

## RECOMMENDATIONS

We recommend considering scale-up of this feasible model with necessary adaptations to hospitals in South Africa and other high HIV burden contexts where gaps in linkage are identified. For effective scale-up, engaging stakeholders, presenting a strong case for change, and co-designing interventions with the Ministry of Health and hospital managers is an essential first step in building confidence in the model. An implementation guide could assist in reducing any need for intensive quality improvement processes if hospital teams choose to adopt this model. Implementation could help minimize readmissions, loss to follow-up, morbidity, and mortality among hospitalized and recently discharged PLHIV. Hospitals serve as important entry points for identifying newly diagnosed and unlinked individuals, especially men, older patients, and those with advanced HIV and comorbidities, such as TB. Task-shifting using dedicated, trained in-hospital teams of lay counselors and NIMART-trained nurses can provide comprehensive HIV-related services. Where such resources are unavailable, a single case coordinator could be assigned to liaise with existing staff to support completion of all necessary linkage steps. Ensuring post-discharge linkage and management is vital to prevent readmissions and improve health outcomes.^14^^,^^19^ Using a single, unified electronic database with a unique patient identifier could support hospital to PHC facility linkage and reduce the human resource costs of tracking patient movements between facilities. This approach addresses the needs of hospitalized individuals, facilitating timely ART initiation and continued care despite clinical barriers during hospitalization.

For effective scale-up, engaging stakeholders and co-designing interventions with the Ministry of Health and hospital managers is essential for building confidence in the model.

### Limitations

As with many quality improvement models, challenges were faced in capturing routine monitoring data, resulting in missing information. A total of 263 (4%) individuals had no ART initiation date captured. However, we included them in overall initiation rates because the 28-day follow-up register confirmed ART initiation within the follow-up period. To avoid overwhelming teams with the administrative burden of time-consuming data collection tools, routine data collection was limited to the HTS register, NIMART nurse register, and linkage officer register, as well as reports. Therefore, clinical details, including baseline CD4 counts, reasons for admission, and comorbidities, were not available for analysis. Reasons for noninitiation by 28 days (declined initiation, death, loss to follow-up, or transfer) were incompletely recorded. All patients requiring ART, including newly diagnosed, previously known with HIV but never initiated ART (pre-ART), and previously on ART with current interruption, were considered as a single group “needing ART.” Consequently, we were unable to disaggregate these 3 groups to evaluate differences in linkage outcomes. Should future research be undertaken, we would recommend including this variable in data collection and analysis. Data were also incomplete for those who declined both in-hospital ART initiation and consent for telephonic follow-up.

Implementation challenges affected fidelity across sites, particularly regarding collaboration with hospital management at some sites and consistent staffing. The COVID-19 pandemic limited teams to non-COVID-19 patients, and HIV testing was focused in green zones, potentially missing patients who were admitted to and discharged from COVID-19 wards. Lastly, because of inadequate monitoring and data processes before implementing our model, the only available comparator data came from DHIS reports, which do not disaggregate by gender. Consequently, we were unable to analyze improvements in linkage by gender. Furthermore, hospital 5, the poorest performing facility, faced its own data challenges, as its independent in-house system did not consistently feed data into DHIS. Given that this hospital was the third largest in the group, excluding it from our analysis was not feasible, but its data inconsistencies likely impacted our overall findings.

We chose to disaggregate the top 2 performing hospitals to demonstrate the potential for high linkage rates when quality implementation and robust data reporting are both established.

## CONCLUSION

Through reviewing existing PHC facility-based and hospital-based HIV services, we were able to identify key discrepancies in staff roles and responsibilities to inform a quality improvement process that led to a refined and optimized hospital HIV service model. By combining in-hospital HTS and ART initiation with post-discharge patient support provided by a dedicated team, ART initiation within 28 days reached 74% at 5 hospitals in Johannesburg. The model also strengthened referral pathways between hospitals and PHC facilities, resulting in an overall 87% ART initiation rate. The overall goal of initiating 90% of PLHIV on ART within 28 days was not achieved, emphasizing ongoing challenges managing HIV patients transitioning between hospital and PHC services. This structured follow-up and patient support model helped overcome barriers to ART initiation, which may exist during acute hospital admissions, including clinical complications and psychological readiness.^12^ Continued efforts are needed to develop approaches to improve psychosocial support for patients who remain reluctant to commit to lifelong ART and smooth the transition between hospital and PHC services. The model’s success relied on acceptance and support from the Department of Health, hospital management, and ward-based staff to fully operate. Building strong relationships between the dedicated HIV team and hospital clinical teams proved essential.

This model highlighted the potential for effective interventions to improve outcomes for hospitalized PLHIV. More than half of all hospitalized patients were eligible for and accepted rapid ART initiation, and weekly follow-up supported successful post-discharge initiation in many others. Implementing this model in more hospitals has the potential to improve HIV-related clinical and intervention outcomes.

## Notes

**Peer Reviewed**

**First Published Online:** January 14, 2025.
